# MUC1, MUC2, MUC5AC, and MUC6 in colorectal cancer: expression profiles and clinical significance

**DOI:** 10.1007/s00428-016-1970-5

**Published:** 2016-06-14

**Authors:** Johannes Betge, Nora I. Schneider, Lars Harbaum, Marion J. Pollheimer, Richard A. Lindtner, Peter Kornprat, Matthias P. Ebert, Cord Langner

**Affiliations:** 1 Department of Medicine II, University Hospital Mannheim, Medical Faculty Mannheim: Heidelberg University, Mannheim, Germany; 2 Institute of Pathology, Medical University of Graz, Auenbruggerplatz 25, 8036 Graz, Austria; 3 Department of Medicine II, University Medical Center Hamburg-Eppendorf, Hamburg, Germany; 4 Department of Surgery, Medical University of Innsbruck, Innsbruck, Austria; 5 Department of Surgery, Division of General Surgery, Medical University of Graz, Graz, Austria

**Keywords:** Mucin, Prognosis, Stage II, Immunohistochemistry, Large intestine, Prognostic factor

## Abstract

Mucin glycoprotein expression can be altered during the carcinogenic process. The impact on the prognosis of patients with colorectal cancer (CRC) is controversial. We analyzed tumors from 381 patients for MUC1, MUC2, MUC5AC, and MUC6 expression by immunohistochemical staining, using tissue microarrays. Progression-free and cancer-specific survival were determined using the Kaplan-Meier method. Expression of intestinal mucin MUC2 was lost in 85 (23 %) CRCs, and patients with MUC6-negative tumors showed shorter progression-free survival (PFS, *p* = 0.043). Gastric mucins MUC5AC and MUC6 showed high (>50 %) aberrant expression in 28 (8 %) and 9 (2 %) cases, respectively. High expression of MUC5AC was associated with longer PFS (*p* = 0.055). High expression of MUC6 was associated with 100 % PFS (*p* = 0.024) and longer cancer-specific survival (CSS, *p* = 0.043). MUC1 was expressed in 238 (64 %) tumors and had no impact on outcome. When analysis was restricted to stages II and III, loss of MUC2 was associated with adverse outcome. Overexpression of both MUC5AC and MUC6 significantly predicted favorable PFS and CSS. In conclusion, loss of MUC2 expression proved to be a predictor of adverse outcome, while the gain of aberrant expression of MUC5AC and particularly of MUC6 was associated with favorable outcome in CRC, notably in intermediate stages II and III.

## Introduction

Tumor stage reflected by the AJCC/UICC TNM system is currently the strongest prognostic parameter for patients with colorectal cancer (CRC) and is therefore used as basis for therapeutic decisions [1]. However, patients with tumors of the same pathologic stage may experience substantially different clinical outcomes, especially in intermediate stages of disease. Different patients may thus benefit from different therapeutic and surveillance strategies [2, 3]. Hence, identification of additional prognostic markers might be of interest to improve stratification of patients.

Mucins are high-molecular-weight glycoproteins expressed by epithelial tissues. They have a high content of clustered oligosaccharides, forming a mucosal protection system at the surface of the gastrointestinal tract [4]. Most of the recent progress in understanding mucin biology is related to the characterization of mucin proteins (apomucins) [5]. Mucins can be classified as secreted gel-forming mucins (MUC2, MUC5AC, and MUC6), transmembrane mucins (MUC1), and other mucins that do not fit into either class [5, 6]. Throughout the gastrointestinal tract, epithelial cells commonly synthesize more than one mucin, although a particular mucin type may predominate in an organ-specific manner. For example, MUC2 is characteristically observed in goblet cells of small and large bowel mucosa, whereas MUC5AC and MUC6 are mainly expressed in gastric epithelium and are rarely observed in the normal colon [5–7]. In contrast, MUC1 is expressed on the apical surface of most epithelial cells [8]. Of note, during neoplastic transformation and/or progression, expression of specific mucins may be reduced or the organ specificity lost, while new mucins may be expressed aberrantly.

In colorectal tumors, several mucins have been analyzed, in relation to the adenoma-carcinoma sequence, MUC1 and MUC2 being the best characterized [4, 6–9]. However, data on the clinical significance, particularly the potential prognostic value of mucin expression in colorectal carcinoma (CRC) are limited and contradictory [5, 6, 9, 10].

Therefore, our study aimed to comprehensively assess the prevalence of MUC1, MUC2, MUC5AC, and MUC6 expression in a large cohort of CRC patients, its association with various clinicopathological parameters such as T classification, N classification, lymphovascular invasion, grade, tumor border configuration, mismatch repair (MMR) status, as well as patients’ progression-free and cancer-specific survival.

## Patients and methods

### Case selection

Case selection of our cohort has been described in detail previously [11]. Briefly, 400 CRC patients treated from January 1992 through December 2000 were randomly selected from the CRC database of the Institute of Pathology, Medical University of Graz, Austria. We excluded (1) patients with T1 cancer treated by endoscopic polypectomy, (2) patients that received neoadjuvant chemotherapy, (3) patients with synchronous or metachronous invasive cancers originating from the colorectum or other sites. In total, 381 resection specimens from 400 patients (95 %) were available for pathology review. Of these, 215 were males (56 %) and 166 females (44 %) (ratio 1.3:1) with a mean age of 68.5 years (median 70.1, range 27.6–93.1).

Stage I and II patients did not receive adjuvant therapy, while stage III patients were treated with 5-fluorouracil/folic acid according to the Mayo Clinic regimen [12]. Patients with node-positive rectal cancer received adjuvant radiotherapy. Follow-up chest X-ray and abdominal ultrasound were performed at 6-month intervals for the first 3 years and yearly thereafter. Moreover, laboratory testing (blood count, liver enzymes, CEA, and CA19–9) was performed at 3-month intervals for the first 3 years and 6-month intervals thereafter. Patients with rectal cancer underwent pelvic computerized tomography every 12 months. Patients were followed after resection until death or time of last follow-up. Disease progression was defined as local tumor recurrence or development of distant metastasis.

### Pathological evaluation

Original histopathological slides were independently re-evaluated by two gastrointestinal pathologists (M.J.P. and C.L.). Tumors located in the cecum and transverse colon were defined as right-sided cancers, while tumors located in the left colonic flexure down to the sigmoid colon were defined as left-sided cancers. Tumors located at the rectosigmoid junction or in the rectum were defined as rectal cancers. Tumor stage was assessed according to the AJCC/UICC 2009 issue of the TNM classification [13]. Histological tumor type and tumor grade were established according to the WHO guidelines [14]. The presence of lymph and/or blood vessel invasion was assessed as carcinoma cells present in vessels with an unequivocal endothelial lining (lymphatic invasion) or in vessels with a thick vascular wall and red blood cells in the lumen (blood vessel invasion). Tumor border configurations were evaluated according to the Jass classification (expanding vs infiltrative) [15]. The extent of tumor budding (presence of isolated single cells or small clusters of tumor cells in the stroma at the invasive tumor margin) was assessed on H&E-stained slides in a field in which budding intensity was maximal [16]. The number of budding foci was scored as low grade (<10 budding foci) or high grade (>9 budding foci) [17].

### Immunohistochemistry

A tissue microarray technique was used for immunohistochemical evaluation as described previously [18]. Briefly, tissue microarrays (TMAs) were constructed using a manual tissue-arraying instrument (Beecher, Silver Spring, MD, USA). Between 3 and 14 (mean 5.03, median 5) cylindrical core biopsies, 0.6 mm in diameter, were taken from different sites of each tumor and arrayed in a recipient paraffin TMA.

For immunohistichemical staining, 4 μm TMA sections were treated with 1 % H_2_O_2_, subjected to antigen retrieval and subsequently incubated for 30 min with primary antibodies using automated staining systems (Universal Staining System, DakoCytomation, Glostrup, Denmark, or BenchMark, Ventana Medical Systems, S.A, Illkirch CEDEX, France). Details on primary antibodies, dilution, and epitope retrieval are listed in Table 1. The reaction was visualized by the Dako EnVision system detection kit, or the ultraVIEW Universal DAB detection kit (Ventana). Goblet cells in adjacent non-neoplastic colonic mucosa served as positive control for MUC2, slides of gastric cancer, known to express MUC1, MUC5AC, and MUC6 served as positive control for MUC1, MUC5AC, and MUC6. Negative controls included omission of the primary antibodies and incubation with Dako ChemMate Antibody Diluent (code no. S 2022) or Ventana Antibody Diluent (catalog no. 251–018), respectively.

**Table 1:** Antibodies used for immunohistochemical staining

Antibody	Clone	Dilution	Epitope-retrieval	Chromogen	Source
MUC1	Ma695	1:100	HIER	DAB	Novocastra, Newcastle upon Tyne, UK
MUC2	Ccp58	1:50	HIER	DAB	Novocastra
MUC5AC	45 M1	1:100	MW, Tris–HCl urea	AEC	Abnova, Taipei, Taiwan
MUC6	MCN6.01	1:100	MW, Tris–HCl urea	AEC	Neomarker, CA, USA
MLH1	G168–15	1:50	MW, buffer pH 9	DAB	Biocare, Concorde, CA, USA
MSH2	G219–1129	1:50	Buffer CC1 standard	DAB	Ventana, Tucson, AZ, USA
MSH6	BC-44	1:50	Buffer CC1 mild	DAB	Biocare

*AEC* 3-amino-9-ethylcarbazole, *buffer pH 9* target retrieval solution, Dako (S2367), *CC1* cell conditioning 1 (Ventana 950–124 SL), *DAB* 3,3′-diaminobenzidine, *HIER* heat induced epitope retrieval solution (Dako, code no. K 5205), 40 min 98 °C, *MW* microwave

Immunoreactivity of MUC1, MUC2, MUC5AC, and MUC6 was assessed by two investigators (J.B. and N.I.S.), who were blinded to clinicopathologic data. Distinct membranous and/or granular cytoplasmic staining was considered positive. Immunoreactivity was semiquantitatively categorized as “negative” (0 % of tumor cells positive) as “low” (<50 % of tumor cells positive) or as “high” (>50 % of tumor cells positive). Each tumor was scored by assessing the average immunoreactivity of the TMA cores. For validation of staining results obtained from TMA slides, we performed immunohistochemistry on corresponding whole sections in selected cases (compare below).

MMR status was assessed as described earlier, using antibodies directed against MLH1, MSH2, and MSH6 [18]. The loss of immunoreactivity for at least one of the three markers characterized MMR-deficient tumors [19].

### Statistical analysis

Associations with T classification, N classification, AJCC/UICC stage, grade, lymphovascular invasion, MMR status, location, tumor type, tumor budding, and tumor border configuration were analyzed using the chi-squared test or Fisher’s exact test. Cause of death was determined by treating physicians and/or by chart review and was corroborated by death certificates if available. Progression-free/disease-free (PFS) and cancer-specific survival (CSS) were investigated using the Kaplan-Meier method and compared by the log-rank test. For bivariate testing, Cox proportional hazards regression models were performed. Statistical calculations were performed using R version 3.2.0 (R Foundation for Statistical Computing, Vienna, Austria; http://www.R-project.org/) or with SPSS version 20.0 (IBM, Armonk, NY, USA). All reported *p* values were two-sided with significance at *p* < 0.05.

## Results

### Tumor characteristics

We classified 28 (7 %) tumors as pT1, 70 (18 %) as pT2, 218 (57 %) as pT3, and 65 (17 %) as pT4. Lymph node metastases were detected in 165 (43 %) cases. Tumor grades were G1 in 121 (32 %), G2 in 138 (36 %), and G3 in 122 (32 %) cases. Regarding tumor type, 317 (83 %) cases were adenocarcinomas and 44 (12 %) were mucinous adenocarcinomas. The remaining tumors presented with rare histological subtypes such as 13 (3 %) undifferentiated carcinomas, 3 signet-ring cell, 2 medullary, 1 amphicrine, and 1 adenosquamous carcinoma. Lymphovascular invasion was recorded in 158 (41 %) tumors.

**Fig. 1:**
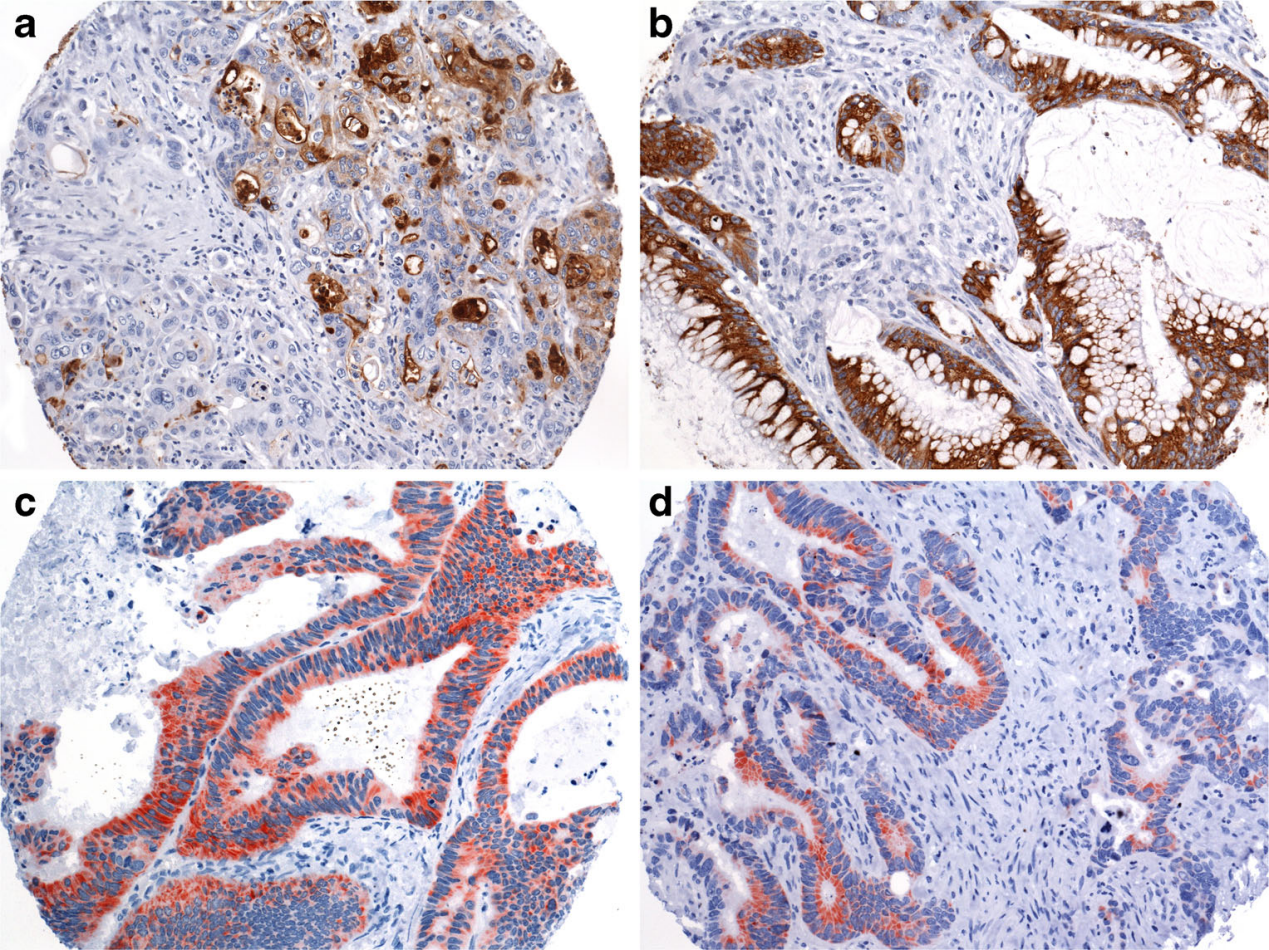
Examples of immunohistochemical staining of MUC1 (×200, **a**), MUC2 (×200, **b**), MUC5AC (×200, **c**), and MUC6 (×200, **d**) using TMA slides

High expression of MUC1 was observed in 46 (12 %) and low expression in 192 (52 %) tumors, while 134 (36 %) tumors were negative for MUC1 (Fig. 1a). High expression of MUC2 was observed in 61 (16 %) and low expression in 225 (61 %) tumors, while expression of MUC2 was lost in 85 (23 %) cases (Fig. 1b). High aberrant expression of MUC5AC was observed in 28 (8 %) and low expression in 155 (42 %) tumors, while 189 (51 %) tumors were negative for MUC5AC (Fig. 1c). High expression of MUC6 was observed in 9 (2 %) and low expression in 99 (27 %) tumors, while 264 (71 %) lacked MUC6 expression (Fig. 1d).

Expression of MUC1 was significantly associated with T and N classification, AJCC/UICC stage, and tumor differentiation and inversely correlated with mucinous adenocarcinoma histological type. MUC2, MUC5AC, and MUC6 were significantly associated with MMR status and tumor border configuration. Additionally, MUC2 expression was associated with mucinous differentiation and inversely correlated with lymphovascular invasion and tumor differentiation. MUC5AC expression correlated with tumor location and mucinous subtype. MUC6 inversely correlated with tumor differentiation and mucinous adenocarcinoma subtype (Table 2). MUC5AC expression significantly correlated with MUC2 and MUC6, while MUC6 expression significantly correlated with expression of MUC1 and MUC5AC (Table 3).

**Table 2:** Association of mucin expression with other pathological variables

		MUC1			MUC2			MUC5AC			MUC6		
		Negative (%)	Low (%)	High (%)	Negative (%)	Low (%)	High (%)	Negative (%)	Low (%)	High (%)	Negative	Low	High
AJCC/UICC stage	I	38 (48)	34 (43)	7 (9)	17 (21)	52 (65)	11 (14)	42 (53)	32 (41)	5 (6)	57 (71)	23 (29)	0 (0)
	II	44 (38)	56 (49)	15 (13)	25 (22)	69 (60)	22 (19)	58 (50)	48 (41)	11 (9)	82 (70)	31 (26)	5 (4)
	III	31 (25)	73 (59)	20 (16)	33 (27)	72 (58)	19 (15)	69 (56)	47 (38)	8 (7)	93 (75)	27 (22)	4 (3)
	IV	21 (39)	29 (54)	4 (7)	11 (20)	34 (63)	9 (17)	22 (41)	28 (52)	4 (7)	36 (67)	18 (33)	0 (0)
	*p*		0.034			0.875			0.615			0.249	
T classification	1–2	44 (47)	41 (44)	9 (10)	18 (19)	63 (66)	14 (15)	50 (53)	39 (42)	5 (5)	69 (73)	26 (27)	0 (0)
	3–4	90 (32)	151 (54)	37 (13)	68 (24)	164 (59)	47 (17)	141 (50)	116 (41)	23 (8)	199 (71)	73 (26)	9 (3)
	*p*		0.040			0.414			0.638			0.209	
N classification	0	88 (43)	95 (46)	23 (11)	44 (21)	129 (62)	35 (18)	106 (51)	84 (40)	18 (9)	147 (70)	58 (28)	5 (2)
	1	22 (27)	53 (65)	7 (9)	19 (23)	54 (66)	9 (11)	44 (54)	35 (43)	3 (4)	60 (73)	19 (23)	3 (4)
	2	24 (29)	44 (52)	16 (19)	23 (27)	44 (52)	17 (20)	41 (49)	36 (43)	7 (8)	61 (73)	22 (26)	1 (1)
	*p*		0.007			0.326			0.666			0.807	
Grade	1–2	80 (32)	139 (55)	33 (13)	59 (23)	179 (70)	17 (7)	132 (52)	107 (42)	15 (6)	167 (65)	81 (31)	8 (3)
	3–4	54 (45)	53 (44)	13 (11)	27 (23)	48 (40)	44 (37)	59 (49)	48 (40)	13 (11)	101 (84)	18 (15)	1 (1)
	*p*		0.045			<0.001			0.240			0.001	
LVI	0	86 (40)	104 (48)	26 (12)	40 (18)	140 (64)	38 (17)	102 (47)	98 (45)	18 (8)	152 (69)	63 (29)	5 (2)
	1	48 (31)	88 (56)	20 (13)	46 (30)	87 (56)	23 (15)	89 (47)	57 (37)	10 (36)	116 (74)	36 (23)	4 (3)
	*p*		0.192			0.041			0.146			0.482	
MMR status	+	120 (35)	183 (53)	45 (14)	83 (24)	214 (61)	52 (15)	184 (53)	142 (41)	24 (7)	243 (69)	98 (28)	9 (3)
	−	13 (57)	9 (39)	1 (4)	2 (9)	12 (52)	9 (39)	6 (26)	13 (57)	4 (17)	22 (96)	1 (4)	0 (0)
	*p*		0.084			0.006			0.024			0.027	
Tumor budding	high	38 (24)	99 (62)	21 (13)	42 (26)	95 (60)	22 (13,9)	86 (54)	60 (38)	12 (8)	109 (69)	46 (29)	3 (1,9)
	low	96 (44)	93 (43)	25 (11)	45 (21)	132 (61)	39 (18,1)	105 (49)	95 (44)	16 (7)	159 (73)	53 (24)	6 (2,6)
	*p*		0.192			0.041			0.146			0.482	
Tumor border	Exp	76 (45)	71 (42)	22 (13)	29 (17)	109 (64)	33 (19)	83 (49)	75 (44)	13 (8)	125 (73)	44 (26)	3 (1,7)
	Inf	58 (29)	121 (60)	24 (12)	57 (28)	118 (58)	28 (14)	108 (53)	80 (39)	15 (7)	143 (70)	55 (27)	6 (2,9)
	*p*		0.084			0.006			0.024			0.027	
Tumor type	Adeno	100 (32)	168 (54)	41 (13)	80 (26)	209 (67)	23 (7)	172 (55)	122 (39)	17 (6)	212 (68)	93 (30)	8 (3)
	Mucinous	27 (63)	15 (35)	1 (2)	0 (0)	7 (17)	35 (83)	12 (28)	24 (56)	7 (16)	37 (86)	5 (12)	1 (2)
	other	7 (35)	9 (45)	4 (20)	6 (30)	11 (55)	3 (15)	7 (35)	9 (45)	4 (20)	19 (95)	1 (5)	0 (0)
	*p*		0.0015			<0.001			<0.001			0.0091	
Location	Right	40 (38)	52 (49)	14 (13)	18 (17)	62 (59)	26 (25)	36 (34)	59 (55)	12 (11)	75 (70)	29 (27)	3 (3)
	Left	35 (33)	53 (50)	19 (18)	25 (23)	71 (66)	12 (11)	57 (53)	40 (37)	10 (9)	75 (69)	29 (27)	4 (4)
	Rectum	59 (37)	87 (55)	13 (8)	43 (27)	94 (59)	23 (24)	98 (61)	56 (35)	6 (4)	118 (73)	41 (26)	2 (1)
	*p*	0.213			0.0421			0.0002			0.7383		

*LVI* lymphovascular invasion, *MMR* mismatch-repair status, *+* proficient, *−* deficient, *Exp* expanding, *Inf* infiltrating

**Table 3:** Interrelations of different mucins expressed in CRC

		MUC2			MUC5AC			MUC6		
		Negative (%)	Low (%)	High (%)	Negative (%)	Low (%)	High (%)	Negative (%)	Low (%)	High (%)
MUC1	0	33 (25)	71 (53)	29 (22)	77 (58)	49 (37)	8 (6)	119 (89)	15 (11)	0 (0)
	<50 %	43 (22)	120 (63)	29 (15)	92 (48)	87 (45)	13 (7)	120 (63)	69 (36)	3 (2)
	>50 %	9 (20)	34 (74)	3 (7)	20 (44)	19 (41)	7 (15)	25 (54)	15 (33)	6 (13)
	*p*		0.0797			0.106			0.001	
		MUC2		0	66 (77)	18 (21)	2 (2)	76 (88)	9 (11)	1 (1)
				<50 %	109 (48)	104 (46)	13 (6)	143 (63)	77 (34)	7 (3)
				>50 %	16 (26)	32 (53)	13 (21)	47 (77)	13 (21)	1 (2)
				*p*		0.001			0.001	
					MUC5AC		0	178 (93)	13 (7)	0 (0)
							<50 %	75 (48)	79 (51)	1 (1)
							>50 %	13 (46)	7 (25)	8 (29)
							*p*		0.001	

**Fig. 2:**
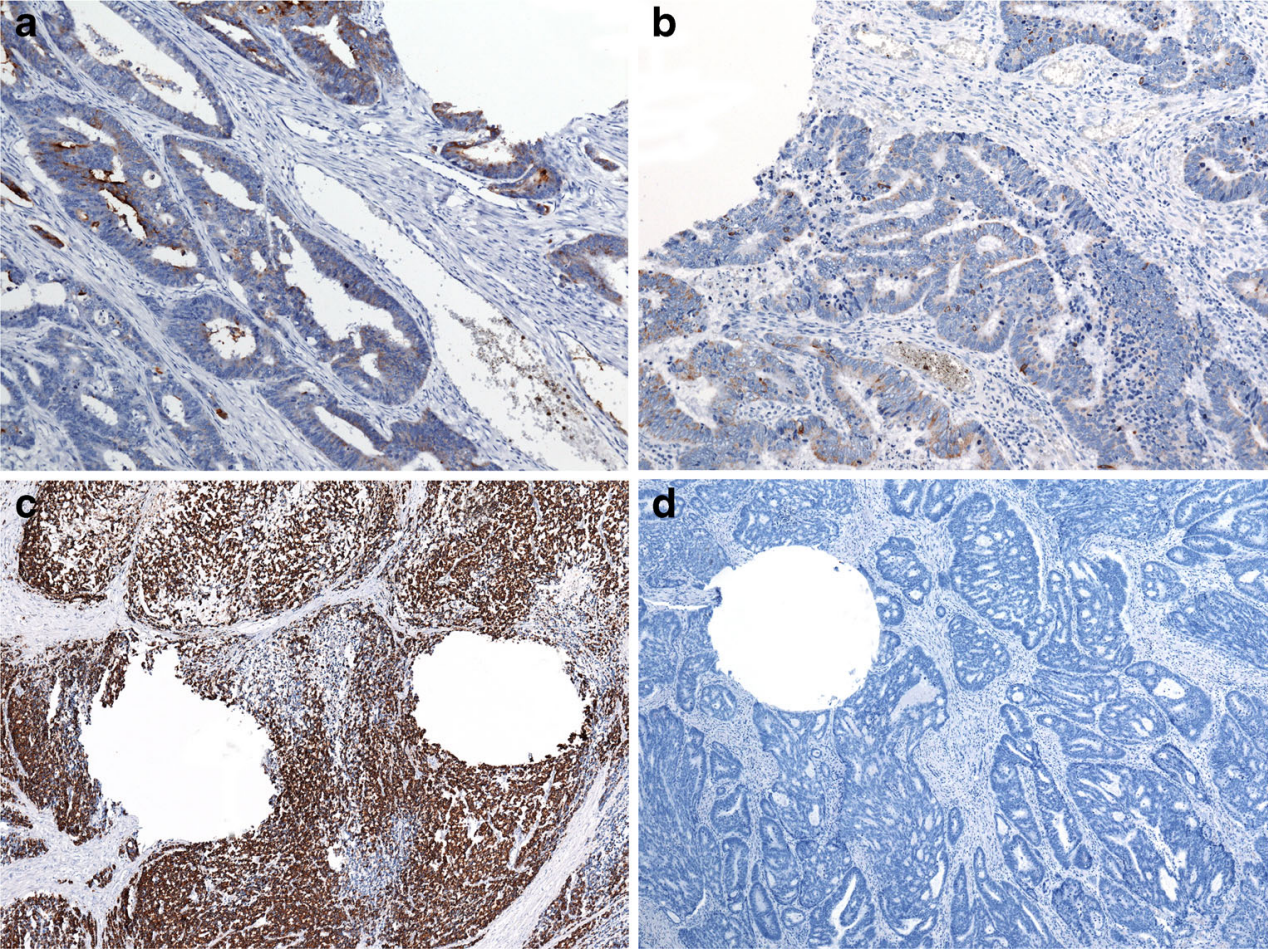
Examples of TMA validation using whole sections (showing identical staining results): low expression of MUC1 (×100, **a**), low expression of MUC2 (×100, **b**), high expression of MUC2 (×40, **c**), and negative staining for MUC5AC (×40, **d**). Roundish tissue defects owing to punch-out of tumor for TMA generation are included in all images

For validation of TMA staining results, a subset of cases with negative and low and high expressions of MUC1, MUC2, MUC5AC, and MUC6 was analyzed on corresponding whole sections. Staining results obtained from TMA slides were confirmed in all analyzed cases (Fig. 2).

### Survival analysis

For 350 out of 381 (92 %) patients, follow-up data were available. Progressive disease was observed in 141 (40 %) patients after a median (mean) follow-up of 45 months (56) (range 1–182). Eleven patients were alive with metastatic disease at the end of follow-up, 118 (34 %) patients died from cancer. Seven patients were without evidence of disease after metastasectomy. Five patients that had presented in poor condition due to advanced disease died within 30 days of surgery. Mean time to progression was 15 months (median 7, range 0–88) [11].

#### MUC1

Disease progression occurred in 43 % of patients with tumors positive (high or low) for MUC1, compared with 36 % of patients with tumors negative for MUC1 (*p* = 0.2). Actuarial 5-year PFS rates were 56 and 64 %, respectively. In addition, 35 % of patients with MUC1-positive and 32 % of patients with MUC1-negative tumors died of disease (*p* = 0.48). Actuarial 5-year CSS rates were 63 and 67 %, respectively.

#### MUC2

**Fig. 3:**
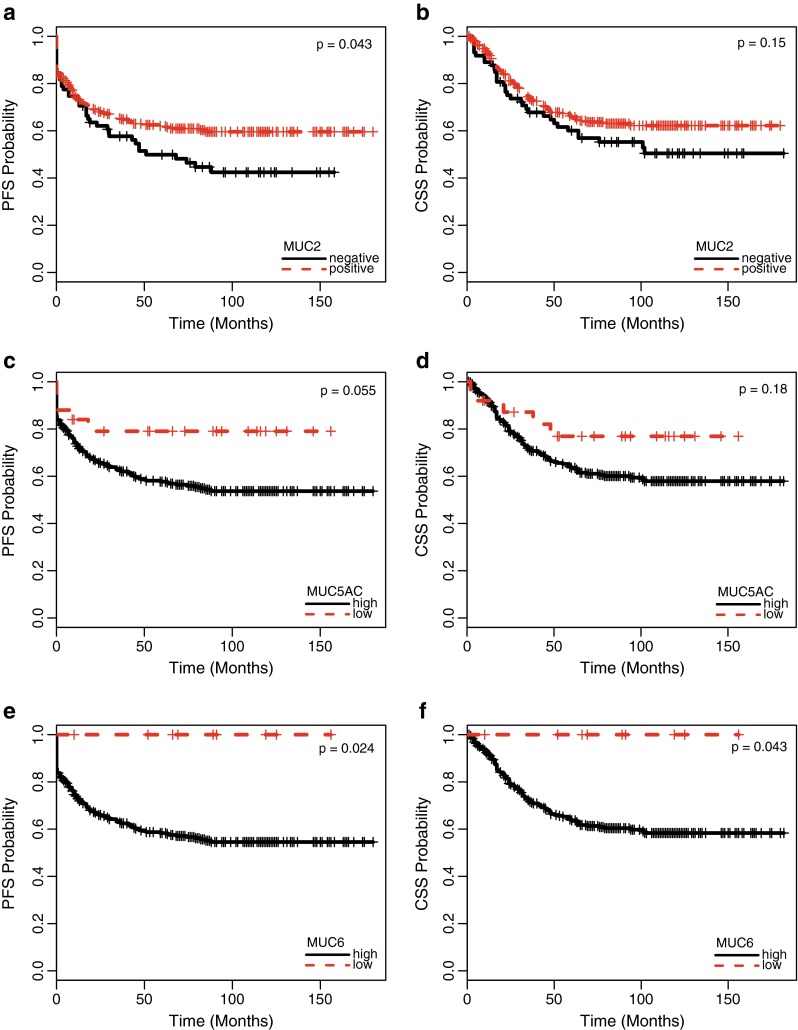
Progression-free (**a**, *p* = 0.043) and cancer-specific (**b**, *p* = 0.15) survival of patients with colorectal cancer related to the extent of MUC2 expression (present vs. absent). Progression-free (**c**, *p* = 0.055) and cancer-specific (**c**, *p* = 0.18) survival of patients with colorectal cancer related to the extent of MUC5AC expression (high vs. low expression). Progression-free (**e**, *p* = 0.024) and cancer-specific (**f**, *p* = 0.043) survival of patients with colorectal cancer related to the extent of MUC6 expression (high vs. low expression)

Disease progression occurred in 52 % of patients with tumors negative for MUC2 compared with 37 % of patients with tumors positive (high or low) for MUC2 (*p* = 0.043; Fig. 3a). Actuarial 5-year PFS rates were 50 and 62 %, respectively. In addition, 42 % of patients with MUC2-negative and 32 % of patients with MUC2-positive tumors died of disease (*p* = 0.15; Fig. 3b). Actuarial 5-year CSS rates were 60 and 66 %, respectively.

#### MUC5AC

Disease progression occurred in 37 % of patients with tumors positive (high or low) for MUC5AC, compared with 44 % of patients with tumors negative for MUC5AC (*p* = 0.46). Actuarial 5-year PFS rates were 63 and 56 %, respectively. In addition, 30 % patients with MUC5AC-positive and 44 % of patients with MUC5AC-negative tumors died of disease (*p* = 0.28). Actuarial 5-year CSS rates were 67 and 62 %, respectively. Of note, the extent of MUC5AC expression was found to be related to disease outcome, that is, only 20 % of patients with high (>50 % of tumor cells) MUC5AC expression experienced disease progression, compared with 42 % of patients with low or absent MUC5AC expression (*p* = 0.055; Fig. 3c). Actuarial 5-year PFS rates were 79 and 58 %, respectively. In addition, 20 % of patients with high MUC5AC expression and 38 % of patients with low or absent MUC5AC expression died of disease (*p* = 0.18; Fig. 3d). Actuarial 5-year CSS rates were 77 and 64 %, respectively.

#### MUC6

Disease progression occurred in 39 % of patients with tumors positive (high or low) for MUC6, compared with 99 out of 243 (41 %) patients with tumors negative for MUC6 (*p* = 0.83). Actuarial 5-year PFS rates were 61 and 59 %, respectively. In addition, 30 % of patients with MUC6-positive and 35 % of patients with MUC6-negative tumors died of disease (*p* = 0.25). Actuarial 5-year CSS rates were 70 and 62 %, respectively. Again, the extent of expression was found to be related to disease outcome, that is, disease progression occurred in 41 % of patients with low or absent MUC6 expression, while none of the nine patients with high MUC6 expression experienced progressive disease (*p* = 0.024; Fig. 3e). Actuarial 5-year PFS rates were 100 and 58 %, respectively. In addition, 34 % of patients with low or absent MUC6 expression and no patient with high MUC6 expression died of disease (*p* = 0.043; Fig. 3f). Actuarial 5-year CSS rates were 100 and 64 %, respectively.

#### Combined analysis

Accordingly, patients with tumors that showed either preserved MUC2 expression and/or high expression of MUC5AC and/or a high expression of MUC6 had longer PFS (*p* = 0.031) but not CSS (*p* = 0.11) than patients lacking those features.

#### Stages II and III

**Fig. 4:**
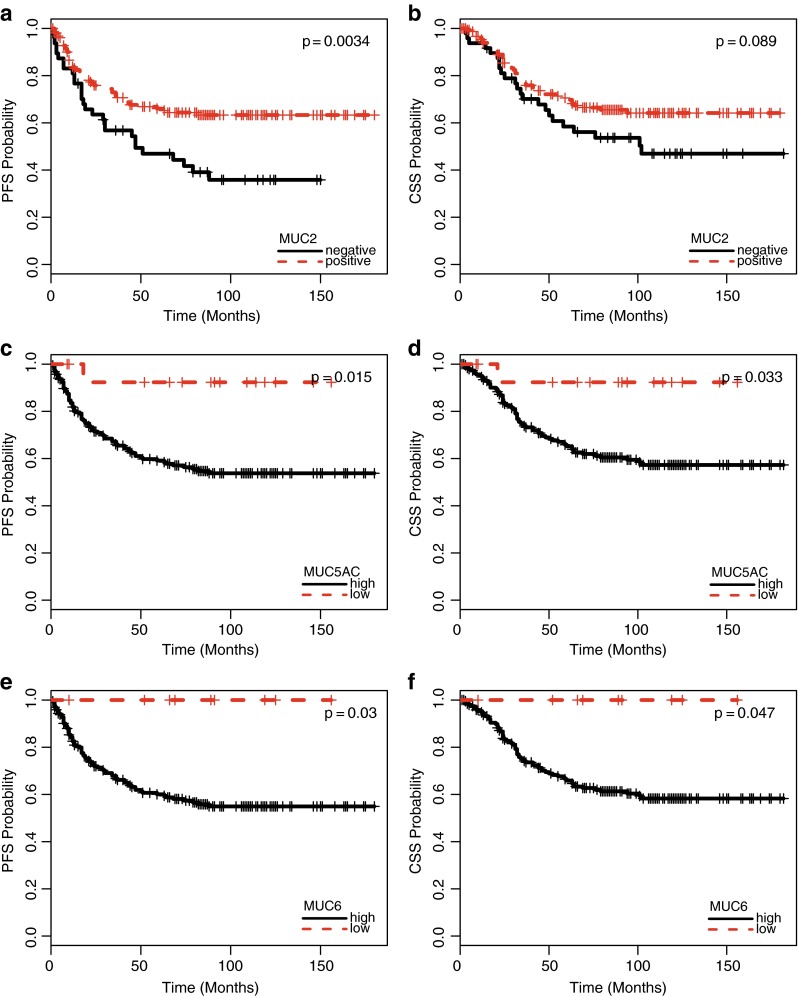
Progression-free (**a**, *p* = 0.0034) and cancer-specific (**b**, *p* = 0.089) survival of patients with stage II and III colorectal cancer related to the extent of MUC2 expression (present vs. absent). Progression-free (**c**, *p* = 0.015) and cancer-specific (**d**, *p* = 0.033) survival of patients with stage II and III colorectal cancer related to the extent of MUC5AC expression (high vs. low expression). Progression-free (**e**, *p* = 0.03) and cancer-specific (**f**, *p* = 0.047) survival of patients with stage II and III colorectal cancer related to the extent of MUC6 expression (high vs. low expression)

Loss of intestinal mucin (MUC2) was also related to shorter PFS (*p* = 0.003, Fig. 4a) and CSS (*p* = 0.089, Fig. 4b), when analysis was restricted to patients with intermediate stages of disease, that is, AJCC/UICC stages II and III. Likewise, gain of gastric mucin (MUC5AC and MUC6) was related to improved outcome. Specifically, high MUC5AC expression predicted favorable PFS (*p* = 0.015, Fig. 4c) and CSS (*p* = 0.033, Fig. 4d), as did high MUC6 expression (PFS: *p* = 0.03, Fig. 4e; CSS: *p* = 0.047, Fig. 4f). Finally, patients with stage II and III disease with tumors that showed either preserved MUC2 expression and/or high expression of MUC5AC and/or a high expression of MUC6 had longer PFS (*p* = 0.0018) and CSS (*p* = 0.062) than patients lacking those features.

#### MMR-positive and MMR-negative patients

Since MMR expression is known to be a confounder of mucin expression, we analyzed MMR positive and negative patients separately. In patients with MMR proficient tumors (*n* = 350), loss of MUC2 was associated with shorter PFS (*p* = 0.024) and CSS (*p* = 0.095). High expression of MUC5AC was related to shorter PFS (*p* = 0.047), while the association of MUC5AC with decreased CSS was not statistically significant (*p* = 0.12). High expression of MUC6 was associated with decreased PFS (*p* = 0.023) and CSS (*p* = 0.042). MUC1 expression showed no impact on PFS (*p* = 0.28) or CSS (*p* = 0.65). In patients with MMR-deficient tumors (*n* = 23), no significant impact on either PFS or CSS was observed.

Additionally, we performed bivariate Cox proportional hazards regression models including MMR status and mucin expression. MUC2 negativity was significantly associated with PFS (HR 1.46, 95 % CI 1.01–2.12, *p* = 0.043), while MMR deficiency was not (HR 1.04, 95 % CI 0.48–2.22, *p* = 0.93). No association with CSS was observed for both markers. High expression of MUC5AC showed a trend toward improved PFS (HR 0.44, 95 % CI 0.18–1.07, *p* = 0.07), while no association of MMR deficiency with PFS was observed (HR 1.04, 95 % CI 0.49–2.23, *p* = 0.92). Again, no association with CSS was observed for both markers. Bivariate Cox regression analyses could not be performed for MUC6 due to low case numbers.

When only stage II and III patients were analyzed in bivariate Cox regression models, presence of MUC2 showed significant association with PFS (HR 2.05, 95 % CI 1.29–2.35, *p* = 0.002), while MMR deficiency did not (HR 1.12, 95 % CI 0.35–3.55, *p* = 0.85). A trend toward shorter CSS was observed for presence of MUC2 (*p* = 0.07), but not for MMR deficiency (*p* = 0.95). High expression of MUC5AC was significantly associated with PFS (HR 0.13, 95 % CI 0.02–0.91, *p* = 0.04), while no association of MMR deficiency with PFS was observed (HR 1.06, 95 % CI 0.35–3.37, *p* = 0.92). High MUC5AC expression showed a trend toward inferior CSS (*p* = 0.06), while no association of MMR deficiency (*p* = 0.89) with CSS was noted.

## Discussion

Mucins have an important function as protective layer for epithelial tissues in the gut and elsewhere in the body. It is well known that during carcinogenesis mucins can be lost or aberrantly expressed in locations where they are not present constitutively. They might be involved in tumor progression and spread. However, the prognostic value of aberrant mucin expression in CRC is controversial [5, 6, 9, 10].

In this study, we show that the loss of intestinal mucin MUC2 predicted adverse outcome, while the gain of gastric differentiation, as documented by aberrant MUC5AC and particularly MUC6 expression: was associated with favorable outcome. In addition, expression of MUC1 proved to be a marker of tumor progression and lymph node metastasis. A significant association between MUC1 expression and survival was, however, not detected in our cohort.

Previous publications have reported impact of MUC1 expression on tumor progression and also on survival [10, 20–24]. For instance, MUC1 expression has been related to higher TNM stage and reduced recurrence-free and overall survival in 206 patients with CRC [22]. Duncan et al. [23] found no association with T or N classification, but also reported a significant reduction of CSS for MUC1-positive tumors. Lugli et al. [10] analyzed a large number of tumors and observed, similar to our data, a positive association of MUC1 with T classification, but not with survival in MMR-proficient tumors. They also found no association with clinicopathological parameters in MLH1-negative cases. Hence, while MUC1 expression appears to be related to tumor stage, its impact on survival remains controversial.

MUC2 is a colonic mucin usually expressed by goblet cells. It is enriched in mucinous adenocarcinoma and can be lost during the carcinogenic process in conventional adenocarcinoma. Similar to MUC1, previous data about the prognostic impact of MUC2 are conflicting. In concordance with our results, Elzagheid et al. [25] found that the presence of MUC2 significantly predicted longer disease-free survival and disease-specific survival in 141 CRC patients across all stages. Accordingly, Lugli et al. [10] reported on a large set of 1420 cases that loss of MUC2 is associated with the presence of lymph node metastasis and with worse survival in both MMR-proficient and MMR-deficient tumors. In another study focusing on stages II and III CRC, Kang et al. [26] likewise noted significantly decreased overall survival when MUC2 expression was lost. However, other publications failed to identify a prognostic impact of MUC2 in CRC [20, 22, 24].

Literature data on the prognostic value of gastric type mucins MUC5AC and MUC6 in CRC are limited. Our study is the first to systematically analyze the prognostic impact of MUC6 expression in CRC. The expression of MUC6 is rare: We observed low expression of MUC6 in 27 % and high expression of MUC6 in only 2 % of patients. However, when MUC6 was present in the majority of cancer cells, affected patients had an excellent prognosis. Therefore, we propose that MUC6 (and MUC2) should be included in the panel of molecular markers applied in prospective studies aiming at risk stratification of CRC patients. Walsh et al. [27] identified MUC6 expression in 33 % of CRCs. According to their and other literature data [28], MUC6 and MUC5AC expression is strongly associated with features of the serrated neoplasia pathway. The impact on outcome was not analyzed in that study. Interestingly, in vitro data generated in a study investigating pancreatic, colorectal, and breast cancer cell lines suggest that MUC6 may inhibit invasion of tumor cells through the basement membrane [29]. This may be a possible mechanism to explain why patients with tumors strongly expressing MUC6 have a favorable outcome.

The aberrant expression of MUC5AC in CRC was more frequent than that of MUC6 in our study. It predicted favorable outcome, and this effect was particularly strong in patients with stage II and III disease. In agreement with our data, a small study analyzing 41 cancer and 41 normal mucosa specimens as well as 21 metastatic lymph nodes also found that patients with tumors expressing MUC5AC had longer disease-free and of overall survival [30]. Another study reported a significant prognostic impact only in poorly differentiated adenocarcinoma, but not in well-differentiated or mucinous-type adenocarcinomas [31]. In a different study, MUC5AC was marginally associated with survival, but statistically significant only in stage IV disease. Hence, our data generally support the limited evidence from literature in that MUC5AC appears to be a significant, but moderately strong prognostic parameter in CRC.

MMR status was significantly associated with the expression of MUC2, MUC5AC, and MUC6, which is in concordance with literature data [27]. In MMR-proficient tumors, loss of MUC2 expression and high expressions of MUC5AC and MUC6 were associated with poor outcome, while no association with survival was detected in patients with MMR-deficient tumors. However, our cohort might not be suitable to prove the lack of impact of mucin expression in MMR-deficient tumors due to relatively small sample size in this subgroup of patients. In bivariate Cox proportional hazards regression models, we were able to confirm the prognostic impact of both MUC2 loss and MUC5AC overexpression in intermediate stages II and III, while MMR status had no impact on outcome. According to a previous study, lack of MUC2 was found to be an adverse prognostic factor in both MMR-proficient and MLH1-negative CRC, but not in presumed HNPCC cases [10]. Data analyzing the prognostic impact of MUC5AC and MUC6, stratified by MMR status, have been lacking so far.

Divergent results in previous studies with respect to the prognostic impact of different mucins, especially MUC1 and MUC2, may be related to the methodology of assessment. We aimed to assess mucin expression in a systematic and easily reproducible fashion in a large number of CRC patients using a high number of tumor tissue cores. However, in other studies, different scoring systems have been used with different cutoffs [10, 20, 22–24, 26, 30]. Also, different antibodies are used with different staining protocols. Moreover, some authors have scored mucin expression specifically at the invasive margin [21, 32]. This makes the comparison of studies difficult. Future (prospective) studies should aim to standardize the methodology of assessment, before testing whether MUC2, MUC5AC, and especially MUC6 expression can be used to stratify patients regarding therapy decisions in routine practice.

Our study has several other limitations. The analysis of microsatellite instability was performed only by immunohistochemical staining of MMR proteins, not by molecular analysis. Furthermore, there are general limitations of retrospective studies, which we tried to control by random selection of patients from a large institutional database and by adherence to strict exclusion criteria. Nonetheless, even considering these limitations, our data strongly suggest that loss of MUC2 as well as aberrant expression of MUC5AC and especially of MUC6 are relevant prognostic indicators in CRC.

In conclusion, we found loss of MUC2 expression to be a predictor of adverse outcome, while gain of aberrant MUC5AC and particularly MUC6 expression was associated with favorable outcome in CRC, especially in intermediate stages II and III. Further prospective studies evaluating adjuvant chemotherapy in stages II and III colon cancer should include MUC2, MUC5AC, and MUC6 expression analysis for patient stratification.
